# Is double-approach surgery and tenodesis without a gastrocnemius flap better for dealing with proximal fibular osteosarcoma?

**DOI:** 10.1186/s12957-018-1364-z

**Published:** 2018-03-27

**Authors:** Jun Wan, Can Zhang, Hong-bo He

**Affiliations:** Department of Orthopaedics, Xiangya Hospital, Central South University, Xiangya Road 87#, Changsha, 410008 Hunan People’s Republic of China

**Keywords:** Double approach, Proximal fibular, Osteosarcoma, Gastrocnemius flap, Tenodesis

## Abstract

**Background:**

Resection of proximal fibular osteosarcoma involving the posteromedial aspect of the fibula is challenging. Reconstruction using a gastrocnemius flap may result in significant lateral instability and abnormal knee movement. Furthermore, postoperative gait may be disturbed by foot drop resulting from scarification of the common peroneal nerve.

**Methods:**

Between January 2011 and December 2013, five patients with proximal fibular osteosarcoma were treated via the double-approach procedure using en bloc resection without a gastrocnemius flap. Simultaneously, all patients received one-stage tenodesis of the anterior tibial and toe extensor tendons. Clinical outcomes, including local tumor recurrence, complications, and functional outcomes, were evaluated.

**Results:**

The mean follow-up duration was 47.2 months (range 42–52 months). No patients experienced local recurrence. The patients’ Enneking functional scores were excellent (80%) or good (20%) at the final follow-up.

**Conclusions:**

In patients with proximal fibular osteosarcoma, the double-approach procedure allows easier and safer en bloc tumor resection with vessel and nerve protection. One-stage tenodesis without a gastrocnemius flap is associated with good functional outcomes.

**Electronic supplementary material:**

The online version of this article (10.1186/s12957-018-1364-z) contains supplementary material, which is available to authorized users.

## Background

As the most common primary malignant bone tumor, osteosarcoma usually involves the metaphyses of the distal femur, proximal tibia, or proximal humerus. In contrast, the proximal fibula is an uncommon site for osteosarcoma. Treatment typically includes preoperative neoadjuvant chemotherapy, surgical resection, and postoperative adjuvant chemotherapy. Joint-sparing procedures with wide surgical margins are the standard surgical intervention.

Described by Malawer, type-II resection is most appropriate for dealing with proximal fibular osteosarcoma. However, if there is marked tumor involvement in the posteromedial region, which is near to the posterior tibial vessels and nerves, amputation is generally selected because of the difficulty of achieving a wide surgical margin [1]. Also, reconstruction using a gastrocnemius flap may result in significant lateral instability and abnormal knee movement [2]. Furthermore, postoperative gait may be disrupted by foot drop resulting from scarification of the common peroneal nerve, which is unacceptable even with ankle–foot orthosis (AFO) [3].

Therefore, we asked the following questions: (1) Is double-approach surgery more appropriate for dealing with proximal fibular osteosarcoma, especially when there is tumor involvement in the posteromedial region? (2) Is one-stage tenodesis an alternative to AFO for dealing with foot drop caused by scarification of the common peroneal nerve? (3) Can better gait be obtained using one-stage tenodesis without a gastrocnemius flap?

## Methods

We performed a retrospective analysis of the clinical medical records, radiography findings, and histologic results of patients treated in our department between January 2011 and December 2013. Patients were eligible for inclusion if they had proximal fibular osteosarcoma histologically proven by core needle biopsy and underwent one-stage en bloc tumor resection via the double-approach procedure and tenodesis of the anterior tibial and toe extensor tendons without a gastrocnemius flap. Patients with multiple lesions of osteosarcoma or metastasis were excluded from the study. The study was conducted in accordance with the Declaration of Helsinki and with approval from the Ethics Committee of our hospital. Written informed consent was provided by all patients for the use of their medical information.

**Fig. 1:**
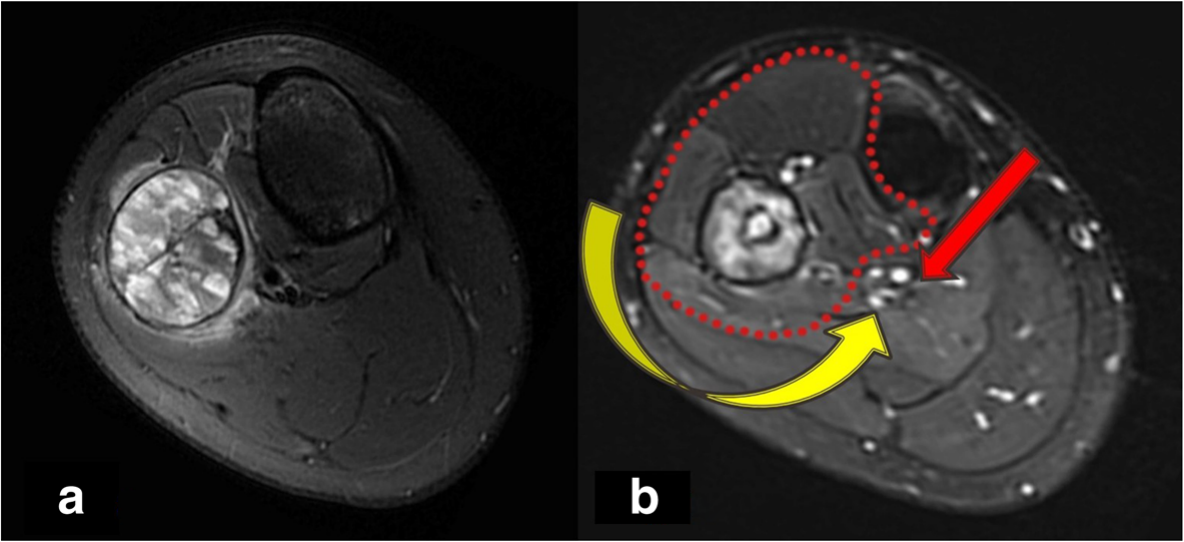
**a** MRI (T2 weight imaging) revealed that the medial-posterior aspect of the proximal fibula was involved by tumor combined with the tibial vessels and nerve. **b** After adjuvant chemotherapy, good result was observed with obvious ossified shell formation and tumor shrink. However, in terms of handling the vessel and nerve, it is difficult to deal from one lateral approach (yellow arrow); we recommended the double approach (adding medial approach, red arrow)

**Fig. 2:**
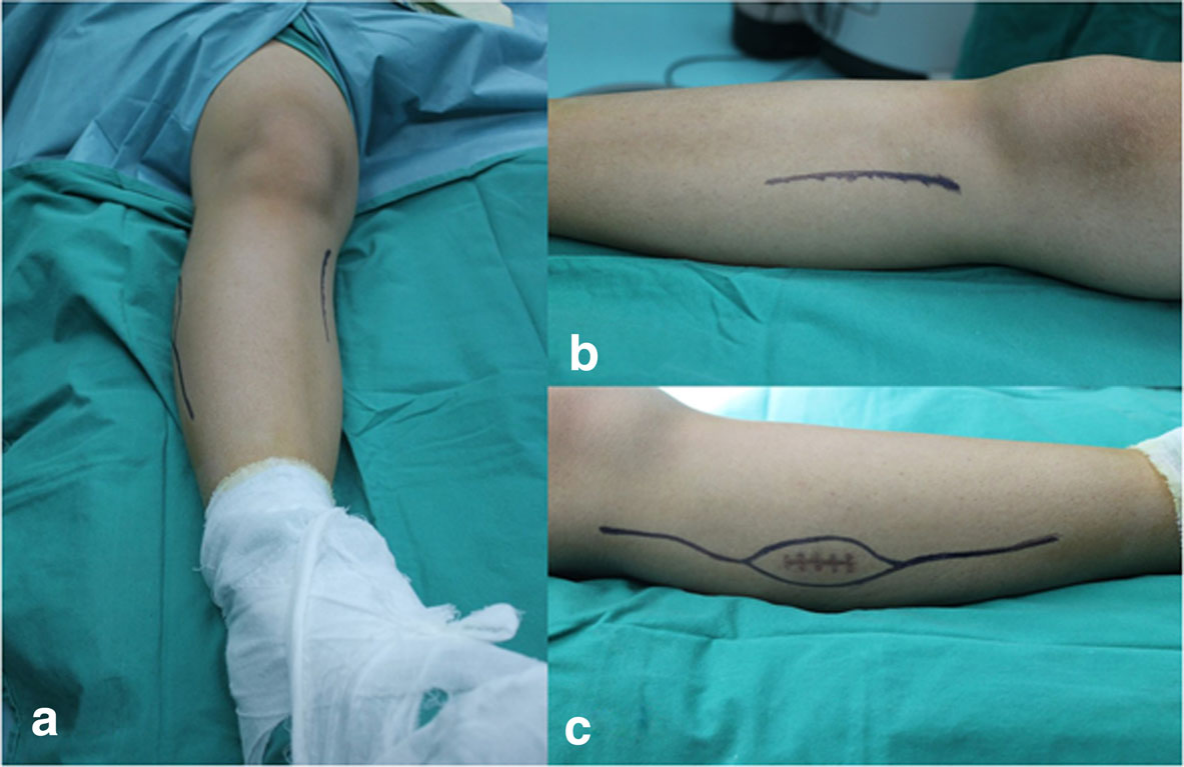
**a** Gross view of the double approach in a patient’s leg. **b** Gross view of the medial approach. **c** Gross view of the lateral approach

All patients had stage-IIB tumors according to the Enneking staging system [4]. A neoadjuvant chemotherapy protocol was administered to all patients (cisplatin 120 mg/m^2^, ifosfamide 3 g/m^2^/day, and doxorubicin 30 mg/m^2^/day). After a two-cycle chemotherapy, the response was evaluated at a multidisciplinary oncologic meeting. All patients exhibited a good response to neoadjuvant chemotherapy before surgery (Fig. 1a, b). Meanwhile, an evaluation of the tibial and fibular arteries was performed using contrast-enhanced computed tomography to eliminate anatomic variations or vascular damage during chemotherapy. Then, modified Malawer type-II resection was conducted to achieve a negative surgical margin (Fig. 2a–c).

**Fig. 3:**
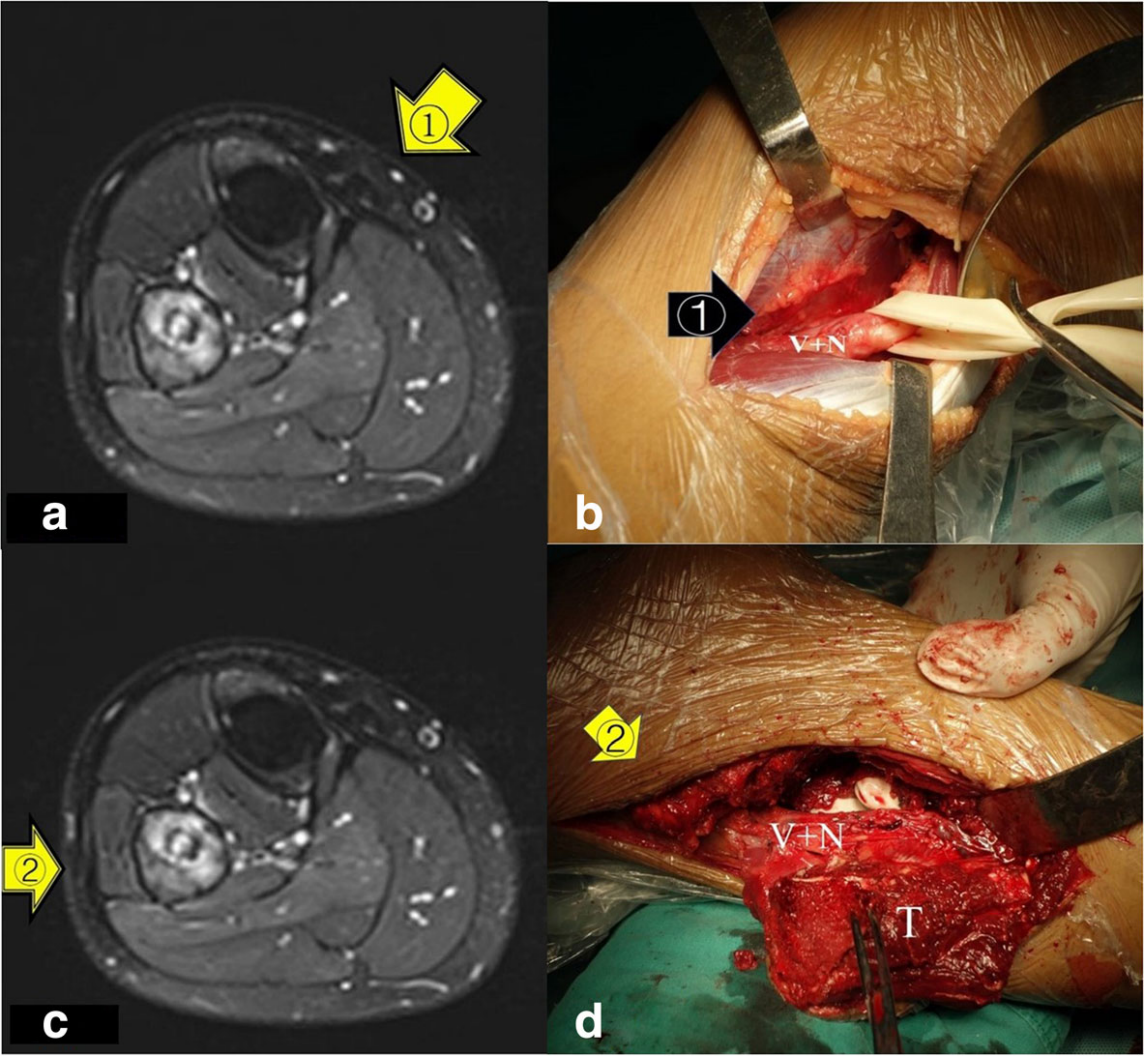
**a**, **b** The first lateral approach (yellow arrow). Through this approach, the anterior tibial artery and the peroneal artery can be ligated easily to secure a safe margin and tumor mobilization; the posterior tibial artery and nerve could be preserved and moderately distracted under direct view (nerve and vessel (N+V)). **c**, **d** The second lateral approach tumor en bloc resection (tumor (T), nerve and vessel (N+V))

**Fig. 4:**
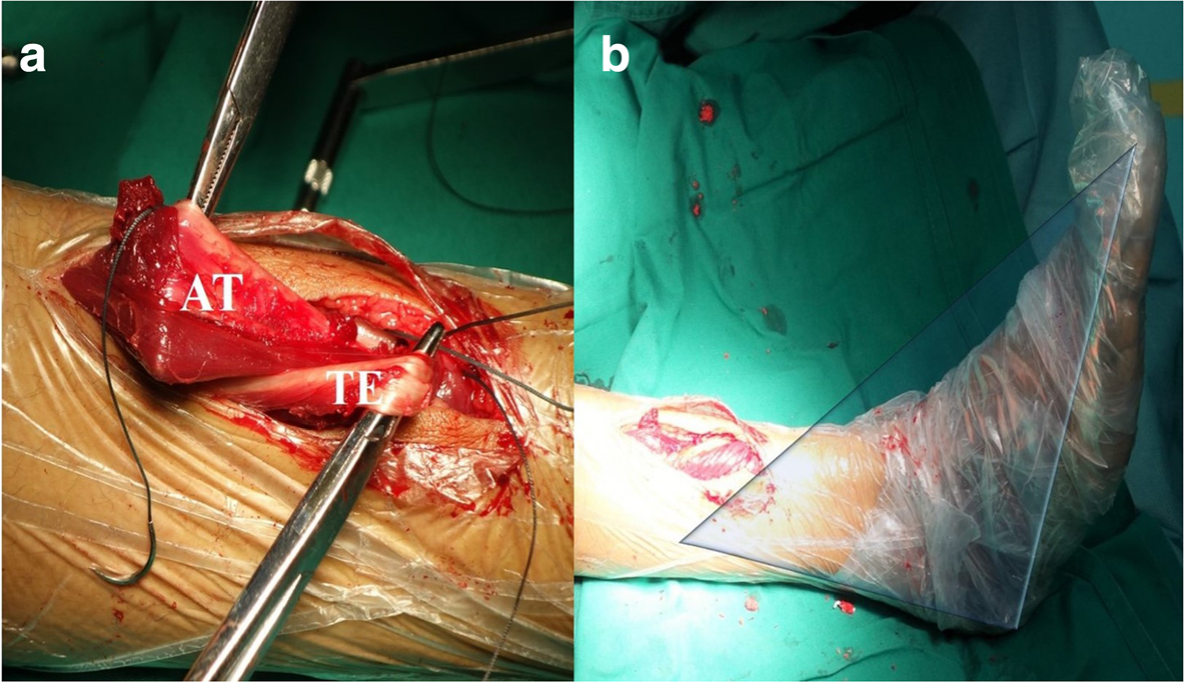
The anterior tibial tendon and toe extensor were stabilized by using anchored screws (**a**) with neutral position of the ankle joint (**b**)

First, in contrast to the standard procedure of Malawer type-II resection, a posteromedial approach was used to expose the posteromedial portion of the tumor, vessels, and nerves in the posterior deep compartment of the leg. Using this approach, ligation of the anterior tibial and peroneal arteries was performed to secure a safer margin and improve tumor mobilization. Then, the posterior tibial artery and tibial nerve were preserved and moderately distracted under direct vision (Fig. 3a, b). Second, a longitudinal incision was made over the proximal fibula to allow excision of the biopsy site. Care was taken to leave a sleeve of normal soft tissue around the tumor in the proximal fibula. Osteotomy of the fibula was performed 2 cm distal to the tumor as indicated by contrast-enhanced magnetic resonance imaging. The common peroneal nerve was sacrificed for achieving sufficient surgical margin (Fig. 3c, d). The cut ends of the biceps femoris tendon and lateral collateral ligament (LCL) were re-anchored to the proximal tibial metaphysis and surrounding capsule for knee stability. A limited compression plate was used to avoid potential facture of the proximal tibia. The wound was closed in lane without a gastrocnemius flap. The anterior tibial and toe extensor tendons were stabilized using anchored screws with the ankle joint in a neutral position (Fig. 4a, b). Finally, histologic examination of the excised specimens was conducted to confirm the diagnosis of osteosarcoma and that the margins were tumor-free.

Two weeks after surgery, adjuvant chemotherapy was initiated and continued for six cycles. Early non-weight-bearing mobilization was allowed by using a hinged knee brace 2 weeks after surgery. Then, full weight-bearing with the brace was permitted 6 weeks after surgery and continued without the brace after 12 weeks. All patients were assessed at 3-month intervals during the follow-up period. At each follow-up visit, routine examinations were performed to test for local recurrence and metastasis. Based on the Enneking functional evaluation scale, the results of functional assessments were recorded as excellent, good, fair, or poor [5].

## Results

**Fig. 5:**
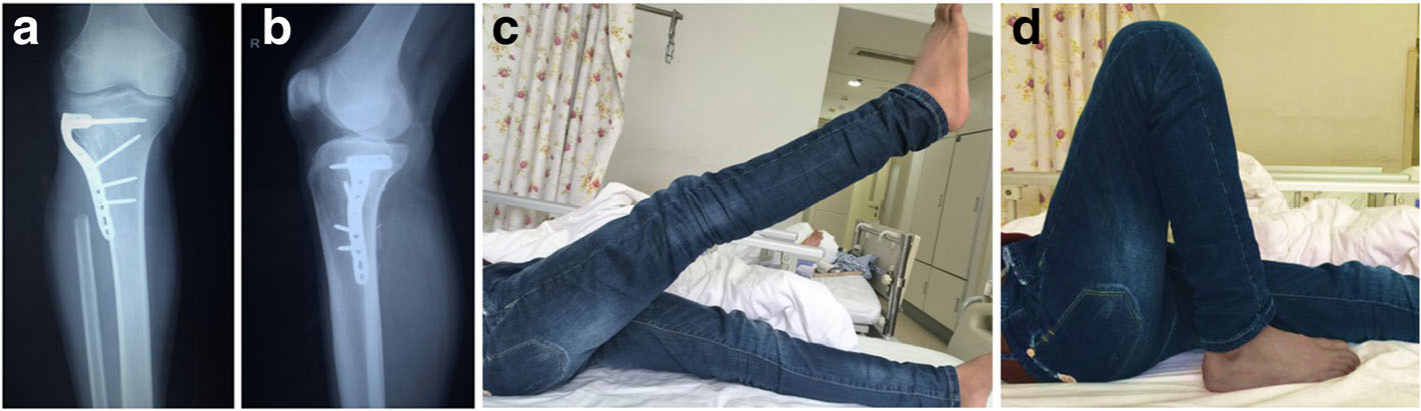
Patient 1, a 16-year-old male, complained pain and limp in his right knee. Radiological reveal suspected proximal fibular osteosarcoma. A double-approach surgery with tenodesis was performed without gastrocnemius flap. At the final follow-up, no local recurrence was presented (**a**, **b**) and the patient was satisfactory with his excellent knee function and gait (**c**, **d**)

Five patients were enrolled in our study (three men and two women) with a mean age of 19.4 years (range 13–37 years). The mean follow-up duration was 47.2 months (range 42–52 months). At the last follow-up, no local recurrence was observed. A suspected lung nodule was found in the left lung of one patient (patient 5) at a follow-up examination at 48 months. One patient (patient 4) required wound debridement for superficial necrosis of the skin. All patients were able to walk without support at the final follow-up. The mean active range of motion of the knee was 143° (range 120–150°) (e.g., patient 1; Fig. 5a–d). The functional results were excellent (80%) or good (20%) at the final follow-up, and no patients were classified as fair or poor (Table 1).

**Table 1:** Patients’ data

Case no.	Age (years)/gender	Length of the tumor (cm)	Longest diameter of the tumor (cm)	Complications+additional procedure	Follow-up (months)	Local recurrence	Metastasis	Enneking score	ROM of knee joint
1	16/M	13.1	6.3	None	44	None	None	Excellent	150°
2	15/M	13.6	5.2	None	52	None	None	Excellent	145°
3	13/F	11.2	3.4	None	42	None	None	Excellent	150°
4	37/F	10.2	3.7	None	48	None	Suspected lung nodule	Good	125°
5	16/M	9.4	4.9	Skin necrosis + debridement	50	None	None	Excellent	145°

*ROM* range of motion

## Discussion

As a result of advances in neoadjuvant chemotherapy, the long-term overall survival of osteosarcoma improved tremendously during the late twentieth century [6]. Limb-sparing surgery has attracted attention because of its enhanced social acceptance and limb function compared with amputation. In proximal fibular osteosarcoma, however, tumor involvement is likely in the posteromedial region, which is near the posterior tibial vessels and nerves; thus, amputation is chosen because of the difficulty of achieving a wide surgical margin.

Recently, several studies have shown that limb-sparing surgery can be applied in patients who exhibit a good response to adjuvant chemotherapy [7]. A study by Xu et al. [8] showed that marginal resection had no negative impact on overall survival or local recurrence in patients who demonstrated a favorable response to chemotherapy. In another study by Kanazawa et al. [9], three patients were treated with intentional marginal resection of proximal fibular osteosarcoma. No local recurrence was observed and good functional results were achieved through preservation of the common peroneal nerve. Reddy et al. [10] analyzed 360 patients with osteosarcoma of the limb treated by two means: resection with close surgical margins or primary amputation. They concluded that amputation offered better local control, but had no clear survival benefit over resection with close margins.

In our case series, no patients experienced local tumor recurrence. This suggests that, even when proximal fibular osteosarcoma occupies a posteromedial position, joint-preserving surgery may be a viable and safe option in patients who exhibit good responses to neoadjuvant chemotherapy.

Malawer et al. [1] described two resection types, the indications of which depend on tumor differentiation: Malawer type-I resection, consisting of marginal resection of the proximal fibula with a small muscle layer, preserving the peroneal nerve and Malawer type-II resection, consisting of wide, extra-articular en bloc resection of the proximal fibula, anterior and lateral muscle groups, and common peroneal nerve. In patients with osteosarcoma, Malawer type-II resection is recommended for a safer surgical margin. However, it is hard to perform this technique through one longitudinal lateral approach if there is tumor involvement in the posteromedial region.

In this study, a modified Malawer type-II resection was used for tumor resection. Our procedure differed in that a posteromedial approach was usually used to expose the vessels and nerves in the posterior deep compartment first. Through this approach, care should be taken to find these structures behind the tumor, which would usually be encountered through the lateral approach in standard Malawer type-II resection. The anterior tibial and peroneal arteries were ligated to secure a safe margin and improve tumor mobilization. Also, the posterior tibial artery and tibial nerve were preserved and distracted under direct vision through this approach, which provided more space for tumor resection.

Surgical treatment of proximal fibular osteosarcoma is challenging because the common peroneal nerve must be sacrificed in order to achieve negative surgical margins, resulting in iatrogenic foot drop. Permanent use of AFO causes muscle atrophy and paresthesia, both of which decrease functional outcomes. Reports in the literature show some loss of function even with use of a functional AFO. Patients also complain about the discomfort of using AFO use.

Tenodesis or tendon transfer is an appropriate alternative to the compensation for peroneal loss and eliminates the need for AFO use [11]. However, compared with simple tenodesis, the procedure of tendon transfer is time-consuming and requires some microsurgical techniques. In our patients, we performed one-stage tenodesis including stabilization of the anterior tibial muscles and toe extensor tendons. The purpose of this was to achieve early foot function without orthosis. All our patients have a satisfactory gait and are not willing to perform two-stage tendon-transfer surgery further (Additional file 1).

En bloc resection of the proximal fibula removes the attachments of the biceps femoris tendon and LCL. The biceps femoris tendon imparts a posteriorly directed dynamic restraint to anterior displacement of the proximal tibia and iliotibial band, providing anterior stability and reducing strain on the anterior cruciate ligament. The LCL provides the predominant resistance to varus rotation loading in the knee [12, 13]. Thus, these lateral supporting structures must be meticulously repaired to prevent postoperative knee instability. Although good function has been reported without ligament reconstruction [14], fixation of the biceps femoris tendon and LCL to the tibial metaphysis is simple. Abdel et al. [15, 16] achieved long-term stability with ligament reconstruction in 112 patients with aggressive benign tumors and 53 patients with malignant fibular tumors. They asserted that, if possible, ligament reconstruction should be regarded as the standard procedure after fibular head resection, especially in young patients.

In Malawer type-II resection, a gastrocnemius flap is usually used for good cover of soft tissue. However, this procedure may cause additional mechanical problems. Kramers et al. [2] noted that abnormal gait could be observed in patients who accepted a gastrocnemius flap procedure. They think the possible reason could be that the knee develops a compensatory mechanism in the swing phase of the gait by increasing peak knee flexion. In our patients, we did not perform this reconstruction, but instead sutured the wound in lane. Only one patient experienced delayed wound healing requiring secondary wound debridement and suturing. We believe that the posteromedial incision made during the double approach reduced some of the tension when suturing the lateral incision, which contributed to wound healing. In selected patients, with precise ligament and soft-tissue suturing, the gastrocnemius flap procedure is not mandatory for soft-tissue reconstruction. In our case series, good functional outcomes of the knee joint were achieved with reattachment of the biceps femoris tendon and LCL and an intact gastrocnemius muscle (Fig. 5c, d).

In consideration of the possibility of bone weakness due to a shortage of soft-tissue coverage, local radiation for potential osteopenia, and pathologic fractures of the lateral tibia [17], a locking compression plate was used to support the lateral wall (Fig. 5a, b).

This study has several limitations. First, it involved a small number of patients with no control group. It was not possible to perform a comparative assessment of safety between the double and single approaches using our patients. Second, there is no consensus regarding the effects on gait of AFO and tenodesis. Third, although the risk of local recurrence was low at the end of the follow-up in our case series, it remains; recurrence may be evident in a longer follow-up period. A minimum follow-up duration of 5 years is necessary to confirm the safety of the procedure.

## Conclusions

In selected patients with proximal fibular osteosarcoma, the double-approach procedure provides more space for local tumor resection and protection of the posterior vessels and nerves. One-stage tenodesis without a gastrocnemius flap achieves good functional results and a satisfactory gait. Although more experience and a longer follow-up are needed, this method may obtain better outcomes and limb function when performing Malawer type-II resection for proximal fibular osteosarcoma.


**Additional file 1:** As presented in this video, patient 2 is satisfied with his gait in his daily life. He is not willing to perform two-stage tendon-transfer surgery once more. (MOV 52410 kb)

